# Risk factors for COVID-19 mortality in hospitalized patients in Bolivia

**DOI:** 10.1016/j.ijregi.2023.10.002

**Published:** 2023-11-01

**Authors:** Jhonny Limachi-Choque, Javier Guitian, Christine Leyns, Miguel Guzman-Rivero, Daniel Eid

**Affiliations:** 1 Centro Universitario de Medicina Tropical (CUMETROP), Faculty of Medicine, Universidad Mayor de San Simon, Cochabamba, Bolivia; 2 Unidad de Epidemiologia, Corporación del Seguro Social Militar (COSSMIL), Cochabamba, Bolivia; 3 Institute of Biomedical Research and Social Research, Faculty of Medicine, Universidad Mayor de San Simón, Cochabamba, Bolivia; 4 The Royal Veterinary College, University of London, London, United Kingdom; 5 Instituto de Investigaciones en Ciencias Sociales, Facultad de Ciencias Sociales, Universidad Mayor de San Simon, Cochabamba, Bolivia; 6 Department of Public Health and Primary Care, Faculty of Medicine and Health Sciences, Ghent University, Gent, Belgium

**Keywords:** Mortality, COVID-19, Comorbidity, Hospitalized, Blood group, Vaccine, Bolivia

## Abstract

**Objectives:**

This study aimed to ascertain which factors are associated with higher risk of mortality among hospitalized COVID-19 Bolivian patients.

**Methods:**

This retrospective observational study assessed risk factors associated with mortality in patients (n = 549) hospitalized for SARS-CoV-2 infection in a Bolivian hospital between April 6, 2020, and August 18, 2022.

**Results:**

The results provide evidence of association between male sex (odds ratio [OR] = 1.6, 95% confidence interval [CI] 1.06-2.6), older age, 51-61 years-old (OR = 5.2, 95% CI 2.2-12.6), 62-70 years-old (OR = 8.7, 95% CI 3.7-20.5), >70 years-old (OR = 16.9, 95% CI 7.1-39.9), and blood group A (OR = 1.9, 95% CI: 1.1-3.4) with higher mortality risk. The strong association between mortality and relatively young age, may be due to high frequency of undiagnosed comorbidities. Vaccination was associated with a reduction in mortality only when time period of hospitalization was not adjusted for.

**Conclusion:**

Among hospitalized patients in Bolivia male sex, older age, and blood group A are associated with higher mortality risk. Mortality risk increased markedly from a relatively young age and decreased in parallel to the uptake of the vaccination program. However, the gradual reduction in mortality can also be due to improved patient management and changes in natural immunity and virulence of circulating strains as the pandemic progressed.

## Highlights

• Bolivia had one of the highest COVID-19 excess mortality despite its young population.
• In the hospitalized, older age and mortality are associated from relatively young age.
• Blood group A, rare in highland indigenous people, is associated with higher mortality

## Introduction

Since its emergence in 2019, SARS-CoV-2 has caused high mortality worldwide, although with large differences between countries. Excess mortality, the most widely accepted metric of mortality since it does not depend on the availability and use of tests, has been highest in Eastern Europe, the Caucasus region of Asia, and South America [1], [2], [3]. Peru, Ecuador, and Bolivia are among the top 25 countries in excess mortality since the start of the SARS-CoV-2 pandemic [4]. However, there is a paucity of data describing key features of the SARS-CoV-2 epidemic, such as mortality in hospitalized patients, in these countries. The high impact of the SARS-CoV-2 epidemic in countries such as Bolivia is particularly striking due to its young population structure (7.62% of the population were 65 or above in 2021 [5]).

Several demographic and comorbidity factors have been found to be associated with COVID-19 mortality in hospitalized patients [6], [7], [8]. A meta-analysis of 122.192 hospitalized patients published after the first wave of SARS-CoV-2 infections gave a pooled mortality of 18.88%. The study identified associations between mortality and age ≥ 65 (relative risk [RR] 3.59), male gender (RR 1.63), intensive care unit admission (RR 3.72), obesity (RR 2.18), hypertension (RR 2.08), diabetes (RR 1.87), cardiovascular disease (RR 2.51), and cancer (RR 2.31) [9]. Although US studies observed the highest prevalence of comorbidities in COVID-19 patients, the mortality was highest in Eastern European and Latin American countries [10]. In Bolivia, the relationship between comorbidities and mortality has only been studied in small samples, over a short period of time and analyzing few risk factors; a more comprehensive analysis is lacking [11,12]. Furthermore, the Bolivian context is especially interesting in view of its exceptional geographic and ethnic diversity, with 62% of the population identifying as indigenous as well as the high mortality reported despite its relatively young population [13].

The SARS-CoV-2 pandemic urged for a reorganization of health services, transforming some hospitals into COVID-19 centers [14], [15], [16]. This was also the case for the Bolivian COSSMIL (Corporación del Seguro Social Militar) hospital, in the city of Cochabamba. This is a second level hospital for members of the armed forces and their families. Patients presenting to the hospital were assessed, based on the severity of their condition they were either hospitalized or referred to a higher-level hospital for further treatment. The COSSMIL hospital restructured its facilities to provide assistance to an important number of COVID-19 patients. The criteria for the diagnosis of COVID-19 and treatments changed based on the availability of tests and specific drugs. This hospital is especially interesting since it received patients from different socio-economic strata and regions of the country. Therefore, this study describes the demographic characteristics and health related factors associated with COVID-19 mortality in the COSSMIL hospital in Cochabamba, Bolivia. The objective of the study is to ascertain which factors are associated with higher risk of mortality among hospitalized COVID-19 Bolivian patients. It is expected that the results of the study could provide insights into potential differences with other countries that may explain the particularly high mortality due to COVID-19 in Bolivia and other Andean countries and contribute to improved prevention and management of severe SARS-CoV-2 infection in Bolivia and the Andean region.

## Methods

### Study design and population

**Figure 1:**
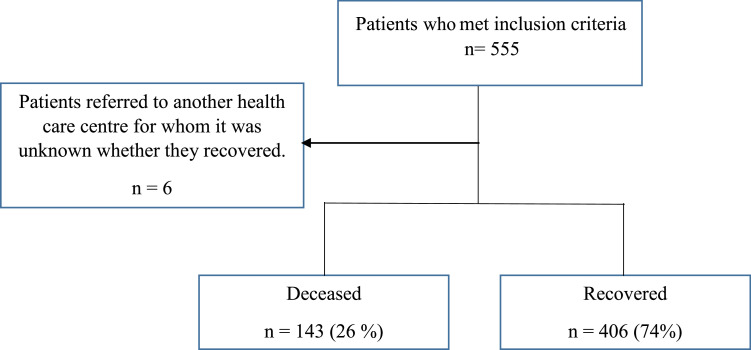
Flowchart describing the recruitment of study participants as part of the retrospective single center study of risk factors for mortality due to COVID-19 in hospitalized patients in Bolivia.

A retrospective single center study, based on the analysis of medical records from patients with confirmed COVID-19, hospitalized between April 06, 2020, and August 18, 2022, in COSSMIL hospital was performed. SARS- CoV-2 infection status was ascertained by means of a positive polymerase chain reaction (PCR) (n = 314) or positive rapid antigen test (n = 95) or pulmonal computerized tomography (CT) scan with ground glass opacities on the image with negative PCR (n = 24) or pulmonal CT scan with ground glass opacities without PCR or rapid antigen test (n = 116) (Figure 1). Patients hospitalized for reasons other than COVID-19 with a positive SARS-CoV-2 test were excluded.

The management protocol for hospitalized patients along the study period changed as new evidence became available and the disease was better understood.

The entire study period can be split into three sub-periods representing different phases of the pandemic and its control:

i. April 2020 to March 2021 (12 months): the first COVID-19 wave until vaccination became available. Treatment of hospitalized patients during this period was based on the use of ivermectin, azithromycin, corticosteroids, and anticoagulants.
ii. April 2021 to September 2021 (5 months): the second COVID-19 wave, the management protocol for hospitalized patients included remdesivir, corticosteroids, and anticoagulants.
iii. September 2021 to August 2022 (12 months): after the second wave until the end of the study. During this period favipiravir and molnupiravir were additionally used.

Clinical and epidemiological data were drawn from the hospital's electronic medical records and assessed for completeness by the epidemiology and data processing unit. A patient was considered to have been vaccinated when they had received at least one dose of COVID-19 vaccine more than 2 weeks and less than 6 months before hospitalization.

### Study setting

The study was conducted in a second level hospital (COSSMIL) that is part of the army health insurance, located in Cochabamba, Bolivia (16). COSSMIL hospital provides care for 9569 insured patients, most of whom are older patients, and in addition it acts as a referral center for other smaller regional hospitals in neighboring departments. Between 2020 and 2022, many of its facilities were refurbished as part of the contingency plan for the fight against the COVID-19 pandemic, and four intensive care units and 40 inpatient beds were set up. The inpatient beds were used for patients who required both low-flow and high-flow oxygen, with a total of 467 patients treated. In the intensive care unit, where more invasive procedures were performed, 82 patients made use of the four beds.

Hospitalization criteria encompassed respiratory distress, high fever over 38°C, and gastrointestinal issues such as nausea and diarrhea. Radiological signs of pneumonia were also considered for admission, irrespective of comorbidities. Oxygen saturation below 90% was considered but this was not a strict criterion. For severe cases needing immediate intervention, markers included a respiratory rate over 30/minute, a qSOFA score of 2 or more, and SpO2 levels under 93%. Other urgent indicators were a PaO2/FiO2 ratio under 300 mmHg, altered mental status, rapid bilateral lung involvement over 50% within 24-48 hours, ARDS, septic shock, and MODS.

### Identification of comorbidities related to COVID-19, before their hospitalization

The status of each patient included in this study with respect to pre-existing comorbidities was determined based on registration of the disease in their medical history, laboratory results, and imaging studies. The comorbidities considered and their definition for purpose of this study are presented in Table 1. Other comorbidities initially considered included asthma, heart disease, thyroid disease, and chronic obstructive pulmonary disease but they were not included due to small number of cases and / or high proportion of cases that were presumptive without definitive diagnosis.

**Table 1:** Comorbidities considered in the study of risk factors for COVID-19 mortality in hospitalized patients in Bolivia.

Comorbidity	Diagnostic criteria
Cancer	Metastatic or invasive cancer stages II to IV
End-stage kidney disease	A history of kidney transplantation or currently on dialysis
Diabetes	Diagnosis and treatment in medical history
Hypertension	Diagnosis and treatment in medical history
Obesity	Body mass index≥30 at time of hospitalization

In the cohort under investigation, blood type was universally available for all included patients. The requirements of military insurance coverage mandate blood typing as a standard part of initial enrollment evaluations. Therefore, blood type data were readily accessible for each participant*.*

### Statistical analysis

Potential risk factors of interest in this study included age, sex, comorbidities for which evidence of an association with COVID-19 mortality has been found elsewhere, blood group and vaccination.

Descriptive statistics were performed for all variables of interest and presented using frequency tables and percentages. The variable age was categorized into four levels based on the quartiles of the distribution: <51, 51-61, 62-70, and >70. The extent to which hypothetical risk factors are associated between them was assessed by means of chi-squared test of association and risk ratios obtained from contingency tables for each pair of risk factors.

Univariable associations between each hypothetical risk factor and mortality were assessed by means of univariable logistic regression.

Adjusted associations were assessed by means of multivariable logistic regression. All variables presented in Table 2 were included in the model. In formulating this model, we assume that none of the potential risk factors acts as mediator for the effect of another risk factor. The likelihood ratio test was used to compare models with age included as categorical variable or as a linear trend. To our knowledge, except for vaccination, it is unlikely that relevant confounders for the associations of interests have been omitted from the analysis. A potential concern for assessing the effect of vaccination is the uneven distribution of vaccinated patients across the study period and potential changes (improvements) in the management of hospitalized patients as the pandemic progressed. To address this, a second multivariable model was built including the time period as a covariate. Three time periods were included: period 1 (April 2020-March 2021) corresponding to the first wave of the pandemic in Bolivia, period 2 (April 2021-August 2021) corresponding to the second wave of pandemic in Bolivia, and period 3 (September 2021-August 2022) after the second wave.

**Table 2:** Descriptive statistics and results of univariable logistic regression for the association between hypothetical predictors of COVID-19 mortality in hospitalized patients in Bolivia (n = 549 patients hospitalized).

Variables	Group (n)	% deceased	Odds ratio	95% confidence interval
Sex	F (181)	20%	—– 1.57	—– 1.03-2.41
	M (368)	29%		
Age	≤50 (131)	5%	—–	——
	51-61 (140)	22%	5.04	2.13-11.90
	62-70 (139)	30%	7.67	3.30-17.82
	≥71 (139)	45%	14.68	6.39-33.72
Blood group A	No (458)	24%	—– 1.60	—– 0.98-2.59
	Yes (91)	34%		
End-stage kidney disease	No (525)	25%	—– 2.11	—– 0.91-4.85
	Yes (24)	42%		
Hypertension	No (431)	24%	—– 1.55	—– 1.00-2.42
	Yes (118)	33%		
Obesity	No (476)	25%	—– 1.36	—– 0.80-2.33
	Yes (73)	32%		
Diabetes	No (465)	26%	—– 1.16	—– 0.69-1.95
	Yes (84)	29%		
Cancer	No (533)	26%	—– 0.65	—– 0.18-2.31
	Yes (16)	19%		
Vaccination against SARS-CoV-2 prior hospitalization	No (438)	27%	—– 0.79	—– 0.48-1.29
	Yes (111)	23%		

There were no missing data points for the parameters evaluated in this study.

All statistical analyses were performed using R version 4.2.1.

### Ethical consent

The Ethics Committee of the COSSMIL hospital (code 2022/002) approved the study.

## Results

The complete medical records and health insurance enrollment were obtained and analyzed from 549 hospitalized patients with COVID-19. Of these, 143 (26.05%) died, with a median time from hospitalization to death of 8 days. The remaining 406 patients (73.95%) survived, with a median time from hospitalization to discharge of 8 days. During the first period, 64 (31%) of hospitalized patients died, during the second period 66 (26.72%) of hospitalized patients died and during the third period, 13 (13.68%) of hospitalized patients died. Mortality therefore went markedly down after the second wave of the pandemic.

### Descriptive statistics and univariable analysis

Descriptive statistics and univariable associations between the factors under study and mortality are presented in Table 2.

In the univariable analysis, male sex, older age, and hypertension are associated with a higher mortality risk.

### Multivariable analysis

Comparison of the model with age as categorical variable vs age as linear trend provided weak evidence that the first model was a better fit to the data (Deviance chi square = 4.8493 with 2 df, P=0.089). Furthermore, inspection of the standard errors of the estimates from both models only showed very minor improvements in precision in the model with age as linear tread. Therefore, the model with age as a categorical variable was selected. The results from this model are presented in Table 3.

**Table 3:** Results of the multivariable logistic model for the association between selected potential risk factors and COVID-19 mortality in hospitalized patients in Bolivia (n = 549 patients).

Variable	Odds ratio	95% confidence interval	*P*
Sex • Female • Male	—– 1.685	—– 1.065-2. 666	0.0258
Age category • < 51 • 51-61 • 62-70 • > 70	—– 5.283 8.726 16.947	—– 2.201-12.680 3.700-20.578 7.187-39.960	0.0002 <0.0001 <0.0001
Blood group • Other groups • Group A	—– 1.992	—– 1.160-3.422	0.0125
Hypertension • No • Yes	—– 1.008	—– 0.605-1.680	0.9746
Diabetes • No • Yes	—– 0.925	—– 0.519-1.648	0.7916
End-stage kidney disease • No • Yes	—– 1.230	—– 0.491-3.083	0.6587
Cancer • No • Yes	—– 0.417	—– 0.107-1.622	0.2069
Obesity • No • Yes	—– 1.591	—– 0.864-2.930	0.1363
Vaccination against SARS-CoV-2prior hospitalization • No • Yes	—– 0.568	—– 0.330-0.977	0.0411

The results of the multivariable analysis provide strong evidence of association between male sex, older age, and blood group A with higher mortality risk. On the other hand, mortality risk in hospitalized patients who had been vaccinated is nearly half that of non-vaccinated patients, supporting a protective effect of the vaccine on mortality risk among Bolivian hospitalized patients. However, caution should be taken when interpreting the effect of vaccination as vaccinated patients were mostly seen toward the end of the study. None of the patients hospitalized in period 1 had been vaccinated while this was 26% for the patients hospitalized in period 2 and 48% for those hospitalized in period 3. The effect of vaccination in this model can at least in part be due to improvements in management of hospitalized patients as the pandemic progressed. The results of the model adjusting for period (Table 4) are broadly consistent with those of the previous model except for vaccination, for which the adjusted model fails to provide evidence of an effect while being hospitalized in period 3 is associated with a much lower mortality risk.

**Table 4:** Results of the multivariable logistic model for the association between selected potential risk factors and COVID-19 mortality in hospitalized patients in Bolivia (n = 549 patients), adjusting for period of admission.

Variable	Odds ratio	95% confidence interval	*P*
Sex • Female • Male	—– 1.63	—– 1.02-2.6	<0.05
Age category • < 51 • 51-61 • 62-70 • > 70	—– 5.34 8.84 17.82	—– 2.21-12.86 3.73-20.96 7.5-42.34	<0.001 <0.001 <0.001
Blood group • Other groups • Group A	—– 1.82	—– 1.05-3.16	<0.05
Hypertension • No • Yes	—– 0.99	—– 0.59-1.66	0.97
Diabetes • No • Yes	—– 0.92	—– 0.51-1.65	0.78
End-stage kidney disease • No • Yes	—– 1.41	—– 0.54-3.65	0.47
Cancer • No • Yes	—– 0.42	—– 0.1-1.66	0.21
Obesity • No • Yes	—– 1.71	—– 0.91-3.22	0.091
Vaccination against SARS-CoV-2 prior hospitalization • No • Yes	—– 0.72	—– 0.39-1.33	0.3
Time period • April 2020-March 2021 (first wave) • April 2021-August 2021 (second wave) • September 2021-August 2022	—– 0.97 0.34	—– 0.6-1.57 0.16-0.72	0.91 <0.01

### Bivariate associations between the risk factors of interest

Caution is warranted when interpreting point estimates as the confidence intervals are relatively wide because of the limited sample size and co-occurrence of exposures. Bivariate associations between hypothetical risk factors for COVID-19 mortality among hospitalized patients in Bolivia are presented in supplementary Table S. The values presented in each cell are the risk ratio obtained from a contingency table with the variable in the row as “exposure” and variable in the column as “outcome” and the *P*-value from chi square test of association (in brackets).

## Discussion

This study represents one of the first systematic evaluations of the risk factors for COVID-19 mortality in Bolivia, a country which despite its young population structure experienced one of the highest excess mortalities worldwide during the COVID-19 pandemic [4,5]. The overall hospital mortality for the period between April and August 2020 in this study was 36.6%, below the 49.71% mortality observed in a similar study in Peru [17], but far above the 18.88% mortality obtained in a meta-analysis for the same period [9]. Almost half of the patients included in this study were hospitalized during the second wave of the pandemic. This higher burden of the second wave was also seen in Brazil [18].

The multivariable analysis allows estimation of the association between each factor and hospital mortality controlled for the other variables in the model. Evidence of an association with mortality was found for older age, male sex, and vaccination, which is broadly in agreement with previous studies in other countries [9,19,20]. Older age has repeatedly been identified as the strongest predictor of mortality in patients hospitalized for COVID-19 [21]. Interestingly, our study establishes a strong association between mortality and relative young age between 51 and 61 (vs ≤50 years; age = 51-61 years-OR = 5.28 [95% CI, 2.20-12.68]), which is far younger than the age range established in other studies, which identified 60 (vs <40 years; age = 60-69 years-adjusted hazard ratio = 1.89 [95% CI, 1.08-3.32]) [22], 65 (vs ≤65 years; age >65 years-RR 3.59 [95% CI, 1.87-6.90]) [8], or even 70 (vs; age ≥70 years-OR = 4.68 95% CI, 4.02-5.45]) [20,21], as older age risk factor. This finding could be related to the more frequent presentation of undiagnosed comorbidities (e.g., diabetes) at younger age in low-and middle-income countries such as Bolivia, where it has been estimated that over 80% of people with diabetes are younger than 65 years, compared to only 44% in high-income countries [22]. In our study, almost half of the hospitalized patients older than 70 years (45%) died.

Male patients were over-represented among the hospitalized patients included in this study (67% male vs 33% female) and they were also more likely to die according to the results of the multivariable model (OR = 1.685; 95% CI: 1.065-2. 666). This finding is in agreement with studies in other countries, where it has consistently been found that men were at higher risk of hospital mortality [6,9,18,23]. Dyslipidaemia (46% vs 39%), a history of cardiac disease (39% vs 30%), presence of two or more comorbidities (39% vs 33%) [21], hypertension (62.1% vs 59.6%), diabetes (39.2% vs 36.0%), renal failure (22.3% vs 18.1%, *P* <0.001%), and liver disease (5.9% vs 4.5%, *P* <0.001%) [24] have been seen as more prevalent in men compared to women. In the model used in this study the effect of sex is adjusted for age, hypertension, diabetes, renal failure, and obesity.

Regarding SARS-CoV-2 vaccination, there was a clear inverse association with mortality among the study population of hospitalized patients, but it is not possible to separate the effect of vaccination itself with the effect of other changes introduced to the management of patients as well as changes in natural immunity of hospitalized patients and in virulence of circulating strains as the pandemic progressed. Our criteria for considering a patient as vaccinated was relatively lenient (anyone receiving at least one dose between 6 months and 2 weeks before hospitalization) and Bolivia has made use of a range of vaccines not all of them considered to be highly effective [25]. Should more strict criteria be considered, the effect of vaccination at preventing mortality in hospitalized patients would likely be higher.

Our results provide strong evidence of an association between blood group A and COVID-19 mortality, independent of age, sex, and the main comorbidities. Although an association between severity of COVID-19 infection and blood group was suggested in previous studies [26,27], it is noticeable that in addition to sex, age, and vaccination, blood group is the only other variable associated with mortality in our study. It is possible that the lack of highland genetic ancestry plays a role in this result. The Aymara highland South American population has almost exclusively blood group O, as well as 70% of the Quechua highland population [28], [29], [30]. Another finding that supports this hypothesis is that despite a lower level of obesity, participants with blood group A have a tendency toward higher levels of chronic kidney failure, which could be due to a reduced adaptation to altitude. Cochabamba is situated at an altitude of 2500 meter above sea level and some of the patients referred to the study hospital live in altitudes above 3500 meters.

## Limitations

A limitation of this study is that it took place in a single second level hospital, questioning its generalizability to the rest of Bolivia. Furthermore, the retrospective nature of the study limited our access to pre-recorded variables only. However, the authors’ opinion is that there are no obvious unmeasured confounders that could be responsible for the identified associations of age, sex, and blood group with mortality risk. Evaluation of the effect of vaccination is limited by the potential concurrent introduction of the vaccine and other changes in the management of patients. The relatively small size of the study limits the precision of the estimates and makes particularly important that lack of evidence of association in this study is not interpreted as evidence of lack of association.

## Conclusion

Among hospitalized patients in Bolivia male sex, older age, and blood group A are associated with a higher mortality risk. Age markedly increased the risk of mortality among hospitalized Bolivian patients from a relatively young age. Mortality risk steadily decreased during the study period. This decrease goes together with the uptake of the vaccination program in Bolivia. However, the reduction in mortality can also be at least in part due to enhancement in the management of patients and changes in natural immunity and virulence of circulating strains as the pandemic progressed.

These findings can serve as red flags to identify patients at increased risk of serious COVID-19-associated outcomes at the time of hospitalization in Bolivia and potentially in other countries in the region.

## Declarations of competing Interest

The authors have no competing interests to declare.

## Appendix Supplementary materials

Image, application 1
