# Supplementary material for: Risk factors for COVID-19 mortality in hospitalized patients in Bolivia
Source: IJID Reg. 2023 Nov 1;9:95–101. doi: 10.1016/j.ijregi.2023.10.002 (PMC10643226; doi:10.1016/j.ijregi.2023.10.002)
Supplement: Supplementary file 1 [file mmc1.docx]

**Table S.** Results of bivariate associations between hypothetical risk factors for COVID-19 mortality among hospitalized patients in Bolivia.

|  | Age* | Blood group A | Hypertension | Diabetes | Cancer | Chronic kidney failure | Obesity | Vaccination |
| --- | --- | --- | --- | --- | --- | --- | --- | --- |
| Sex | 1.029  (0.798) | 0.907  (0.715) | 0.827  (0.310) | 0.723  (0.143) | 1.082  (1) | 2.459  (0.130) | 0.944 (0.908) | 1.163 (0.484) |
| Age |  | 0.887 (0.608) | 2.138 (<0.001) | 1.494 (0.070) | 2.49 (0.16) | 2.016  (0.156) | 1.190 (0.510) | 1.473 (0.036) |
| Blood group A |  |  | 1.153  (0.588) | 1.184 (0.615) | 1.678 (0.563) | 2.072  (0.157) | 0.451 (0.058) | 1.174 (0.548) |
| Hypertension |  |  |  | 3.166 (<0.001) | 2.191 (0.203) | 7.305  (<0.001) | 2.851 (<0.001) | 1.480 (0.048) |
| Diabetes |  |  |  |  | 0.791 (1) | 3.954  (<0.001) | 2.236 (0.001) | 1.447 (0.103) |
| Cancer |  |  |  |  |  | 1.448  (1) | 0.938 (1) | 2.242 (0.039) |
| End-stage kidney disease |  |  |  |  |  |  | 1.959 (0.156) | 1.930 (0.058) |
| Obesity |  |  |  |  |  |  |  | 2.396 (<0.001) |

* For purpose of this analysis, age was categorized as <60 vs. >60
